# The relationship between psychological distress and multiple tender points across the adult lifespan

**DOI:** 10.1016/j.archger.2015.10.012

**Published:** 2016

**Authors:** Deborah Brown, Matthew Mulvey, Lis Cordingley, Amir Rashid, Michael Horan, Neil Pendleton, Rosie Duncan, John McBeth

**Affiliations:** a Arthritis Research UK Epidemiology Unit, University of Manchester, UK; b School of Medicine, University of Manchester, UK

**Keywords:** Tender points, Anxiety, Depression, Ageing

## Abstract

Multiple tender points are common in the population and, in studies of mid-life adults, are strongly associated with high levels of psychological distress. Whether this relationship occurs in older adults is unclear.

This cross-sectional study investigated whether high levels of psychological distress would be associated with a high tender point count and whether the relationship would be moderated by age. Three thousand three hundred and seventy-nine individuals were mailed a questionnaire which included the Hospital Anxiety and Depression (HAD) scale, the Pain Catastrophising Scale (PCS), the Brief Illness Perception Questionnaire (Brief IPQ), and the Pittsburgh Sleep Quality Index (PSQI). A random sample of approximately 10% of subjects who returned the questionnaire undertook a physical assessment, including a manual tender point count assessment.

A total of 2385 (71%) subjects completed the questionnaire, of whom 798 (33%) were invited to take part in the physical assessment and 290 (12%) participated. Of the 290 participants the median age was 64 years (range 34–97) and 63% were female. The median HAD score was 9 (IQR 5–14) and the median number of tender points was 3 (range 0–7).

Increasing HAD score was positively and significantly associated with tender point count, but this relationship was not moderated by age. In a final multivariable model, sex, HAD score and PSQI score were independent predictors of multiple tender points.

Psychological distress was associated with multiple tender points independent of age. Psychological distress and trouble sleeping were important, potentially modifiable factors associated with the outcome.

## Highlights

• Greater psychological distress is associated with higher tender point count.
• Age does not change the relationship once co-morbidities are accounted for.
• Poor sleep quality is associated with high tender point count.
• Female sex is also associated with high tender point count.

## 1 Introduction

Tender points are body sites where pain is commonly induced on the application of firm pressure. Although a threshold number of tender points has been part of the American College of Rheumatology definition of fibromyalgia (Wolfe et al., 1990), they are also common in the general population, with one study finding a median of 4 tender points (Croft, Schollum, & Silman, 1994), and another finding that the mean number of tender points was 1.8 in participants with no pain and 6.2 in those with chronic, widespread pain (Wolfe, Ross, Anderson, Russell, & Hebert, 1995).

As well as being more common in people with more widespread pain (Croft, Burt, Schollum, Thomas, & Macfarlane, 1996), a high tender point count is also associated with greater disability. Tender point count was found to correlate significantly with physical disability in female care workers (Lundberg & Gerdle, 2002). In older people multiple tender points have been found to be associated with mobility limitations. A population-based study of older participants (aged 70–97 years) found that higher tender point count was significantly associated with poorer mobility (Eggermont, Shmerling, & Leveille, 2010).

Studies primarily including mid-life adults in the general population have shown strong relationships between high levels of psychological distress and multiple tender points (McBeth, Macfarlane, Benjamin, Morris, & Silman, 1999; Petzke, Gracely, Park, Ambrose, & Clauw, 2003). Whether this association is apparent in older adults is unclear. A large (*n* = 62344) community-based study on participants aged 20–89 years found that depression as measured by the Hospital Anxiety and Depression (HAD) scale increased with increasing age (Stordal, Mykletun, & Dahl, 2003). A study on rheumatology patients found that tender point count increased with age, reaching a maximum at age 70 (Wolfe & Cathey, 1985). Therefore there is evidence that both depression and tender point count increase with increasing age, but their relationship in older adults is unclear.

The aim of this study was to test the hypotheses that high levels of psychological distress would be associated with a high tender point count and that the relationship would be moderated by age.

## 2 Methods

This cross-sectional analysis was part of the Pain Across the Adult Life Span (PAALS) population-based cohort study. All subjects were contacted by post and asked to complete a questionnaire, and a stratified random sample of subjects who had supplied full pain data formed a sub-group, which received a physical assessment including tender point count and screening for osteoarthritis (OA) in the hands, hips and knees. The PAALS study received full ethical approval from the North West 8 Local Research Ethics Committee (10/H1013/29) and the Research Ethics Committee of the University of Manchester.

The subjects were recruited from two sources. The first was a random sample recruited from the general practice registers of three areas in the north west of England in 2001 (Gupta et al., 2007), the second was a non-random sample recruited via newspaper, radio and television advertisements in Newcastle-upon-Tyne and Greater Manchester during the period 1983–1994 (Rabbitt et al., 2004).

### 2.1 Questionnaire

Participants were asked whether they had experienced pain for one day or longer in the past month. Those responding positively were asked if they had been aware of the pain for 3 months or more and identified the painful areas by shading on blank body manikins (back, front, left and right sides). Participants were classified into one of three groups based on their pain response: the “no pain” group, the “CWP” group consisted of individuals who satisfied the American College of Rheumatology criteria for chronic widespread pain (CWP) used in the 1990 definition of fibromyalgia (pain in contralateral body quadrants, above and below the waist, and on the right and left hand sides of the body, which has been present for at least 3 months) (Wolfe et al., 1990), and the “some pain” group were those reporting pain which did not meet this definition. Pain status was used to stratify the subjects for random selection to the physical assessment sub-group, and in stratification of the sample weighting.

Anxiety and depression in the past week were measured using the Hospital Anxiety and Depression (HAD) scale (Zigmond & Snaith, 1983). The HAD contains a seven-item depression sub-scale and a seven-item anxiety sub-scale. A total measure of psychological distress can be obtained by adding the two scores, giving a total between 0 and 42, with higher scores associated with a higher probability of having an anxiety or depressive disorder (Zigmond & Snaith, 1983). Only the total score was used in the present study; because there is significant co-existence of anxiety and depression in older people (Bains, Scott, Kellett, & Saxon, 2014) general psychological distress was felt to be the most relevant measure here.

Sleep quality was measured using the Pittsburgh Sleep Quality Index (PSQI) (Buysse, Monk, Berman, & Kupfer, 1989). This instrument measures sleep quality under seven headings, which can be combined in a PSQI global score between 0 and 21, with 0 indicating no difficulties, and 21 severe difficulties in all areas of sleep quality.

The Pain Catastrophising Scale (PCS) was used to assess pain catastrophizing (Sullivan, Bishop, & Pivik, 1995), and illness beliefs specifically related to pain were assessed with the Brief Illness Perception Questionnaire (Brief IPQ) (Broadbent, Petrie, Main, & Weinman, 2006). PCS scores can be divided into 3 sub-scales: rumination, magnification and helplessness, which were combined into a PCS total score between 0 and 50, with 0 indicating no pain-related catastrophising and 50 the most severe pain-related catastrophising. The Brief IPQ is an eight item scale, each item assessing beliefs as follows: consequences, timeline (chronic, acute), personal control, treatment control, illness identity, concern, understanding and emotional response. Each Brief IPQ item was rephrased to ensure participants responded to each according to their beliefs about pain rather than any other co-morbidity. Items were scored from 0–10, with some having reversed scoring. The items were not designed to be combined into a total score.

Subjects were asked which best described their smoking history: “I smoke now”, “I don’t smoke now but I have done in the past” or “I have never smoked”. They were classified into current smokers, ex-smokers and never-smokers. Participants were asked how many alcoholic beverages they had drunk on average per week over the past three months. This was divided into glasses of red wine, white wine, sherry or fortified wine, pints of beer/lager/cider, measures of spirits, and any other alcoholic drinks. The numbers given were summed to give a total, weekly alcohol consumption.

Co-morbidity was assessed using the number of medications participants were taking. A study on older people found that a simple count of medications was of comparable effectiveness to more complex indices of co-morbidity in predicting health care use and mortality in the ensuing year (Perkins, Kroenke, Unützer, Katon, & Hope, 2004).

### 2.2 Physical assessment

Tender point count was determined by using manual pressure of approximately 4 kg at the points defined by the American College of Rheumatology in their 1990 definition of fibromyalgia (Wolfe et al., 1990). Subjects were asked to rate the sensation from each tender point as “pressure”, “discomfort” or “pain”. If the subject rated the point as “pain”, or gave an involuntary flinch or vocalisation, that point was counted as positive. The number of positive responses was summed as tender point count for that subject.

Sub-group participants were also physically screened for osteoarthritis (OA) of the hand, knee and hip. The OA hand assessment used the American College of Rheumatology criteria (Altman et al., 1990). For a positive identification of OA, in addition to pain, aching or stiffness in the hands, subjects needed to have hard tissue enlargement of at least 2 of 10 selected joints (the 1st carpometacarpal joint, 2nd and 3rd proximal interphalangeal joints and distal interphalangeal (DIP) joints of both hands), soft swelling of fewer than 3 metacarpophalangeal joints, and either hard tissue enlargement of at least 2 DIP joints or deformity at one or more of the 10 specified joints. Participants were screened for OA of the knee using the American College of Rheumatology criteria (Altman et al., 1986). For a positive identification of OA in subjects aged 38 years or under, knee pain and bony enlargement of the knee were required. In subjects aged 39 years or older, knee pain, morning stiffness lasting less than 30 min, and bony enlargement of the knee were required. Participants were screened for OA of the hip using the American College of Rheumatology criteria (Altman et al., 1991). A positive identification required hip pain, medial hip rotation less than 15°, and hip flexion of 115° or less. The number of OA sites was summed in the regression analysis.

### 2.3 Statistical analysis

All analyses were carried out on data from subjects who took part in the sub-study. The data were sample weighted using stratification by sex, age group and pain status, to allow data obtained from the sub-group to be applied to the whole study cohort. Descriptive statistics of median and inter-quartile range or percentages were calculated for each of 3 age tertiles.

As 95 of the sub-study participants had some missing data, imputation using the chained equation method was undertaken. Model 1 regressed psychological distress (as measured by the total HAD score) against tender point count, adjusted for age group and sex only. Model 2 included other variables: catastrophising (PCS), pain beliefs (Brief IPQ), sleep quality (PQSI), alcohol consumption, and smoking status. Model 3 also included measures of co-morbidity: total medication count and number of osteoarthritis sites.

Results are presented as *β* coefficients with 95% confidence intervals. For each model, an adjusted *R*^2^ was calculated to indicate the proportion of variance in the outcome variable explained by that model. All statistical analyses were carried out using Stata version 13.1 (StataCorp, 2013).

## 3 Results

**Fig. 1:**
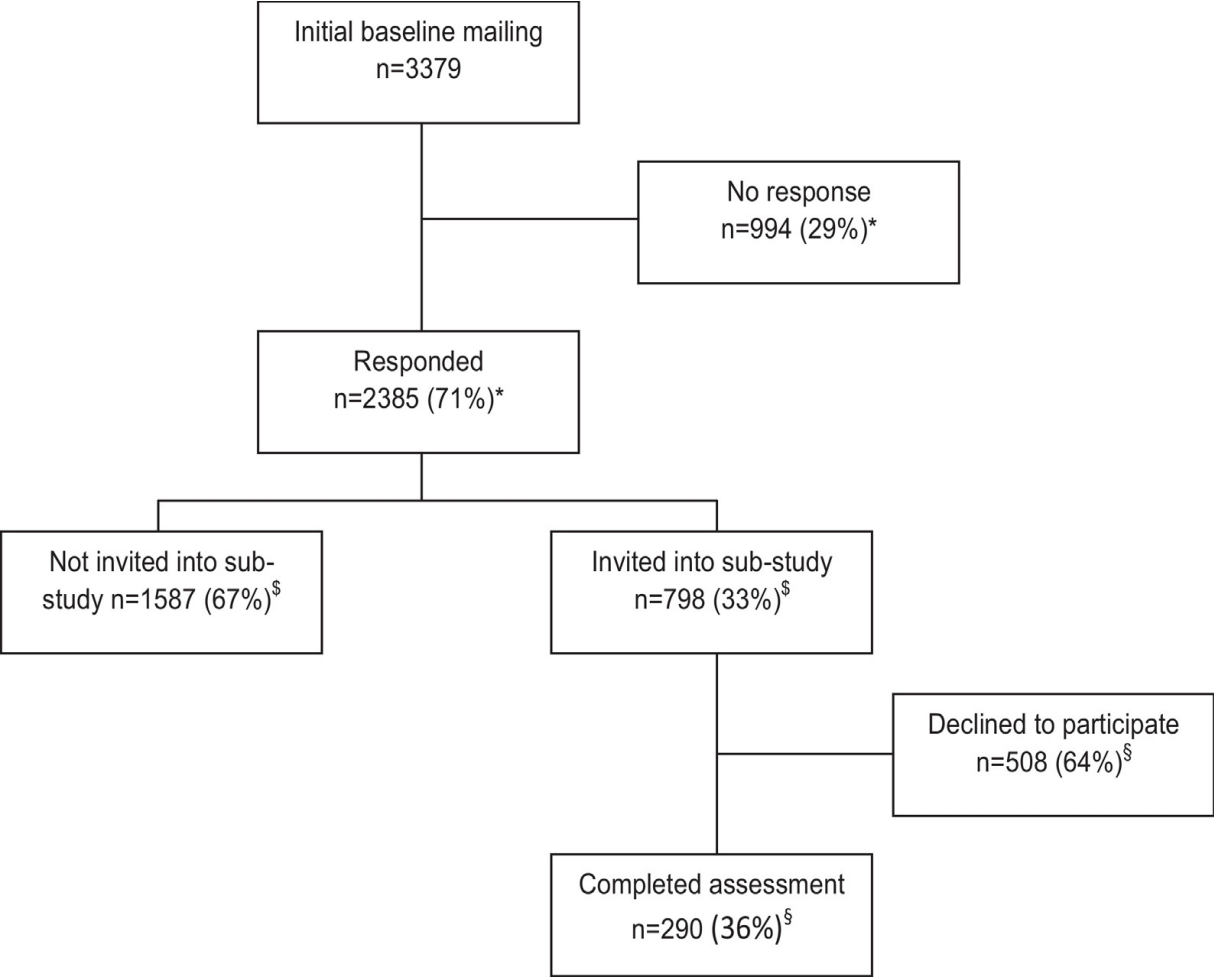
Flowchart of baseline and sub-study participation. ^*^Proportion of initial baseline mailing. ^$^Proportion of those who responded to baseline mailing. ^§^Proportion of those invited to take part in sub-study.

A total of 3379 participants were mailed a questionnaire at baseline, of whom 2385 (71%) returned a questionnaire (see Fig. 1). From the responders, 798 (33%) were randomly selected to be invited to take part in the sub-study and 290 (36%) of these consented and were assessed.

Table 1 summarises the sample weighted data for all variables by age group. Of the 290 sub-study participants the median age was 64 years (range 34–97) and 62.6% were female. The median HAD score was 9 (IQR 5–14) and the median number of tender points was 3 (IQR 0–7).

**Table 1:** All variables by age group.

Variables	All (*n* = 290)	Missing data (*n* = )	Youngest (*n* = 73)	Middle (*n* = 118)	Oldest (*n* = 99)	*p*-value
Age (median (range))	64 (34–97)	0	53 (34–57)	63 (58–69)	82 (70–97)	
Sex (% female)	62.6	0	63.6	56.7	67.6	0.26^a^
Tender point count (median (IQR)) (0–18)	3 (0–7)	2	2 (0–6)	2 (0–6)	4 (0–7)	0.26
Psychological distress (HAD) (median (IQR)) (0–42)	9 (5–14)	12	8 (5–15)	8 (5–14)	9 (6–13)	0.82
Catastrophising (PCS) (median (IQR)) (0–50)	6 (2–11)	22	6 (2–9)	6 (2–13)	6 (3–10)	0.61
Sleep (PSQI) (median (IQR)) (0–21)	6 (5–9)	58	7 (5–8)	7 (5–10)	6 (3–8)	0.08
Pain beliefs (Brief IPQ) (median (IQR))						
Consequences (0–10) Concern (0–10) Coherence (0–10) Timeline (0–10) Treatment control (0–10) Personal control (0–10) Identity (0–10) Emotion (0–10)	2 (1–5) 3 (1–5) 2 (0–5) 5 (2–8) 4 (2–7) 5 (3–7) 1 (0–4) 2 (1–5)	7 6 9 8 8 8 12 9	2 (1–5) 3 (1–5) 2 (1–5) 4 (1–7) 4 (1–7) 4 (2–6) 1 (0–5) 3 (1–6)	2 (1–5) 3 (1–6) 2 (0–5) 5 (2–10) 5 (2–8) 5 (2–8) 0 (0–4) 2 (0–5)	2 (1–4) 3 (1–5) 3 (1–5) 4 (2–10) 5 (2–7) 5 (3–8) 1 (0–3) 2 (0–5)	0.99 0.81 0.65 0.41 0.21 0.27 0.61 0.59
Smoking						
Current smoker (%) Ex-smoker (%) Never-smoker (%)	6.4 39.6 54.0	4	13.0 25.7 61.4	4.3 42.1 53.6	3.0.3 48.8 48.2	0.28^a^
Alcohol consumption (units) (median (IQR)) (0–60)	4 (0–9)	2	5 (1–9)	5 (0–11)	3 (0–7)	0.07
Osteoarthritis Hands (%) Right knee (%) Left knee (%) Right hip (%) Left hip (%)	9.0 5.5 5.6 1.3 2.1	0 0 0 0 0	1.3 6.0 6.0 0.0 3.8	7.7 7.3 8.4 3.1 2.7	16.7 3.4 2.5 0.7 0.0	0.01^a^ 0.49^a^ 0.23^a^ 0.32^a^ 0.77^a^
Medication count (median (IQR)) (0–17)	2 (1–5)	0	1 (0–2)	2 (0–5)	4 (2–7)	0.0001

All *p*-values Kruskal–Wallis except ^a^ = Chi-square; HAD, Hospital Anxiety and Depression scale; PCS, Pain Catastrophising Scale; PSQI, Pittsburgh Sleep Quality Index; IPQ, Illness Perception Questionnaire.

It can be seen that only osteoarthritis in the hands and medication count varied significantly between the age groups (*p* < 0.05), whereas the other variables did not. Table 1 is based on complete data, as only a few participants had missing values for any given variable.

Table 2 gives the results of the regression analyses, carried out on imputed, sample weighted data. In Model 1, psychological distress as measured by the HAD scale was statistically significantly, positively related to tender point count. Female as opposed to male sex but not age was significant in this model, which explained 23% of the variance in tender point count. In Model 2, psychological distress was still significantly related to tender point count, although the relationship was attenuated. Female as opposed to male sex, the oldest age group (age 70–97) compared to the youngest (age 34–57), and sleep quality were also statistically significant in this model, which explained 29% of the variance in tender point count. Model 3 in Table 2 included the co-morbidity measures of osteoarthritis count and number of medications taken. The relationship between psychological distress and tender point count was not further attenuated, and female sex and sleep quality were still statistically significant in this model, although age was not. Three osteoarthritis sites (the maximum any single participant had) compared to no OA sites was statistically significant in this model, but medication count was not. This model explained 30% of the variance in tender point count.

**Table 2:** Relationship between tender point count and distress.

	Tender point count (outcome)		
	Model 1 β coefficient (95% confidence intervals)	Model 2 β coefficient (95% confidence intervals)	Model 3 β coefficient (95% confidence intervals)
Psychological distress (HAD) (0–42)	0.28 (0.20, 0.36)	0.19 (0.08, 0.30)	0.19 (0.09, 0.30)
Age			
Youngest (34–57) Middle (58–69) Oldest (70–97)	(referent) 0.50 (−0.9, 1.87) 1.30 (−0.09, 2.5)	(referent) 0.45 (−0.88, 1.78) 1.46 (0.22, 2.70)	(referent) 0.24 (−1.10, 1.59) 0.86 (−0.53, 2.25)
Sex			
Male Female	(referent) 1.99 (0.98, 3.35)	(referent) 1.71 (0.61, 2.80)	(referent) 1.86 (0.77, 2.94)
Catastrophising (PCS) (0–50)		−0.03 (−0.13, 0.07)	−0.03 (−0.13, 0.07)
Sleep quality (PSQI) (0–21)		0.29 (0.07, 0.52)	0.28 (0.05, 0.50)
Pain beliefs (Brief IPQ)			
Consequences (0–10) Concern (0–10) Coherence (0–10) Timeline (0–10) Treatment control (0–10) Personal control (0–10) Identity (0–10) Emotion (0–10)		0.06 (−0.28, 0.39) −0.01 (−0.30, 0.33) 0.03 (−0.15, 0.21) 0.14 (−0.02, 0.30) 0.001 (−0.17, 0.16) 0.01 (−0.19, 0.21) 0.16 (−0.14, 0.47) −0.09 (−0.43, 0.24)	−0.001 (−0.32, 0.32) −0.04 (−0.26, 0.34) −0.05 (−0.13, 0.23) 0.12 (−0.04, 0.28) 0.01 (−0.15, 0.18) 0.01 (−0.19, 0.21) 0.13 (−0.18, 0.44) −0.12 (−0.45, 0.20)
Smoking			
Never-smoker Ex-smoker Current smoker		(referent) 0.25 (−0.80, 1.31) 0.69 (−1.37, 2.75)	(referent) 0.19 (−0.86, 1.23) 0.40 (−1.80, 2.61)
Alcohol consumption (units)			
(0–60)		−0.04 (−0.09, 0.01)	−0.03 (−0.08, 0.02)
Osteoarthritis count			
0 sites 1 site 2 sites 3 sites			(referent) −0.50 (−1.33, 2.33) 0.78 (−1.47, 3.03) 5.00 (0.46, 9.54)
Medication count (0–17)			0.10 (−0.09, 0.28)
Adjusted *R* squared	0.23	0.29	0.30

HAD, Hospital Anxiety and Depression scale; PCS, Pain Catastrophising Scale; PSQI, Pittsburgh Sleep Quality Index; IPQ, Illness Perception Questionnaire.

An interaction term between psychological distress (total HAD score) and age group was then included in this final model (not shown in Table 2). Neither the interaction term between total HAD score and the middle relative to the youngest age group, nor the interaction term between total HAD score and the oldest relative to the youngest age group, were statistically significant in this model.

## 4 Discussion

The relationship between psychological distress and tender point count found in Model 1 persisted, albeit attenuated, in Model 2 when adjusted for cognitive factors, smoking, alcohol consumption and co-morbidities. However, the initial finding of a moderating effect of age did not persist in the Model 3 when co-morbidities were included.

This finding of a positive relationship between psychological distress and tender point count is in keeping with previous research, which has found associations between tender point count and depression (Croft et al., 1994). The value of *R*^2^ increased from 0.23 in Model 1 to 0.30 in Model 3, demonstrating that these additional factors only made a small difference to the proportion of variance explained. The value of *R*^2^ increased from 0.29 to 0.30 between Model 2 and Model 3, showing that the addition of measures of co-morbidity made almost no difference to the amount of variance explained.

The relationship between tender point count and psychological distress was significantly different for the oldest age group when compared to the youngest in Model 2, but not in Model 3 when co-morbidities were included. This is noteworthy as it implies that the age moderation observed in Model 2 was due to the higher level of co-morbidity in the older age group. Table 1 shows a steady increase in medication count with increasing age, and the prevalence of osteoarthritis in the hands similarly shows a statistically significant increase with age. In the Model 3 (Table 2), the relationship between tender point count and psychological distress was significantly different for people having osteoarthritis at 3 sites compared to none, so it would appear that osteoarthritis at multiple sites together with co-morbidities (as described by medication count) have explained the variance in the relationship between distress and tender point count which was attributed to age in Model 2 (Table 2). The implication of these results is that the relationship between psychological distress and tender point count was not significantly different for the three age groups, when co-morbidities were taken into account. This would seem to suggest that treatments targeting distress are equally relevant in disorders characterised by high tender point counts in people of all ages. Further research investigating the effects of psychological interventions on chronic pain disorders, including adults of all ages, are warranted.

Sex was statistically significant in all 3 models. Previous studies have shown that females are more likely than males to have a high tender point count. A study on low back pain patients aged 16–60 years old found that females were significantly more likely than males to have more than 8 tender points (Jensen, Nielsen, & Stengaard-Pedersen, 2010). In a community sample of subjects with a mean age of 53 years, females were found to have a higher median tender point count than males (Croft et al., 1994). It is often found that females have a higher prevalence of depression and anxiety than males. A study on a community-based sample of people aged 65 and over found that females were more likely than males to have more (≥4 out of 8) depressive symptoms (Lohman, Dumenci, & Mezuk, 2014). A community-based study found higher incidence of depression and of anxiety among females than males, but only up to the age of 50 (Faravelli, Alessandra Scarpato, Castellini, & Lo Sauro, 2013). However, none of these studies looked at whether the relationship between tender point count and psychological distress would be different for males and females. In the present study, the β coefficient in the final model was 1.86, indicating that being female rather than male resulted in just under 2 additional tender points for the same total HAD score. These data suggest that treatments targeting distress may be more relevant for women than for men in chronic pain disorders such as fibromyalgia.

Sleep quality was statistically significant in the multivariable and final models. The β coefficient in the final model was 0.28, indicating that for every 4 points increase in the PSQI there would be slightly more than one additional tender point. Poor sleep quality has previously been found to be associated with high tender point count (Croft et al., 1994). The findings in the present study reinforce the role of sleep quality in musculoskeletal disorders, and further work on interventions is required to determine the effects of improving sleep quality.

The PAALS study was the largest study to date to carry out tender point examinations on participants from a general population. The relatively large number of older participants also distinguished the PAALS study from other studies. The median age of those having the tender point examination was 64 years and the range 34–97 years. This included 42 people aged 85 or older, who can be described as “oldest old” (Dini & Goldring, 2008).

The level of response to the questionnaire (71%) was fairly high when compared to other studies (Rolstad, Adler, & Ryden, 2011). There is no *a priori* reason to suppose that the relationships investigated in this paper would be different in the non-responders than in the responders, so the risk of non-response bias is considered low. The participants recruited by advertisement were older on average than those recruited via GP registers. None of the participants, from either of the recruitment sources, were research-naive, but had taken part in several rounds of data collection. This means that they had self-selected and were all effectively “volunteers”. This might make their responses differ from that of the general population, although once again there is no *a priori* reason to believe this would alter the relationships between variables.

Missing data are always an issue in observational studies. The present study used a method of imputation which is recognised as giving reliable results, to overcome the potential bias of using complete cases only. Only a fraction of the whole study cohort underwent the tender point examination, so this data was “missing by design” for most participants. The use of sample weighting allowed the tender point data to be applied to the whole cohort.

The number of participants in the sub-study was limited by the resources available. Although the number of participants on whom tender point examination was carried out was large compared to other studies, it was small enough to limit the number of variable categories which could be used, e.g. age groups, and still have sufficient numbers of participants in each group to have adequate power in the analyses.

## 5 Conclusion

The findings of this study indicate that there is a persisting relationship between tender point count and psychological distress, and that this relationship does not differ at different adult ages once the effects of co-morbidities are taken into account. The implications of these findings are that psychological interventions could be effective for chronic pain disorders in adults of all ages, but interventions for older adults may need to be multi-faceted to address osteoarthritis and other co-morbidities.

## Conflicts of interest

No conflicts of interest are declared for any author.
